# Development of Tinnitus and Hyperacusis in a Mouse Model of Tobramycin Cochleotoxicity

**DOI:** 10.3389/fnmol.2021.715952

**Published:** 2021-09-01

**Authors:** Ryan J. Longenecker, Rende Gu, Jennifer Homan, Jonathan Kil

**Affiliations:** Sound Pharmaceuticals Inc., Seattle, WA, United States

**Keywords:** hearing loss, tobramycin, hyperacusis, tinnitus, ebselen, aminoglycoside – ototoxicity

## Abstract

Aminoglycosides (AG) antibiotics are a common treatment for recurrent infections in cystic fibrosis (CF) patients. AGs are highly ototoxic, resulting in a range of auditory dysfunctions. It was recently shown that the acoustic startle reflex (ASR) can assess behavioral evidence of hyperacusis and tinnitus in an amikacin cochleotoxicity mouse model. The goal of this study was to establish if tobramycin treatment led to similar changes in ASR behavior and to establish whether ebselen can prevent the development of these maladaptive neuroplastic symptoms. CBA/Ca mice were divided into three groups: Group 1 served as a control and did not receive tobramycin or ebselen, Group 2 received tobramycin (200 mg/kg/s.c.) and the vehicle (DMSO/saline/i.p.) daily for 14 continuous days, and Group 3 received the same dose/schedule of tobramycin as Group 2 and ebselen at (20 mg/kg/i.p.). Auditory brainstem response (ABR) and ASR hearing assessments were collected at baseline and 2, 6, 10, 14, and 18 weeks from the start of treatment. ASR tests included input/output (I/O) functions which assess general hearing and hyperacusis, and Gap-induced prepulse inhibition of the acoustic startle (GPIAS) to assess tinnitus. At 18 weeks, histologic analysis showed predominantly normal appearing hair cells and spiral ganglion neuron (SGN) synapses. Following 14 days of tobramycin injections, 16 kHz thresholds increased from baseline and fluctuated over the 18-week recovery period. I/O functions revealed exaggerated startle response magnitudes in 50% of mice over the same period. Gap detection deficits, representing behavioral evidence of tinnitus, were observed in a smaller subset (36%) of animals. Interestingly, increases in ABR wave III/wave I amplitude ratios were observed. These tobramycin data corroborate previous findings that AGs can result in hearing dysfunctions. We show that a 14-day course of tobramycin treatment can cause similar levels of hearing loss and tinnitus, when compared to a 14-day course of amikacin, but less hyperacusis. Evidence suggests that tinnitus and hyperacusis might be common side effects of AG antibiotics.

## Introduction

Aminoglycosides (AG) such as tobramycin are commonly used to treat cystic fibrosis (CF) patients with recurrent pulmonary infections and those infected with multi-drug resistant tuberculosis (Flume et al., 2009). Unfortunately, AGs are ototoxic and can often result in permanent hearing loss (Staecker, 2021). Recent prospective clinical studies demonstrated that a single course of tobramycin can lead to hearing threshold shifts, word-in-noise deficits, and tinnitus, or sound perception in the absence of a sound source (Garinis et al., 2020; Harruff et al., 2020). For those being treated with AGs for chronic conditions, the cumulative ototoxic effects present an enhanced risk of cochleotoxicity (American Speech-Language-Hearing Association, 1994; Garinis et al., 2017; Elson et al., 2020; Hong et al., 2020). While AG-induced hearing loss is now beginning to be well-documented, little is known about the auditory dysfunctions that often accompany hearing loss. However, more research into the prevalence of tinnitus and hyperacusis should be examined in animal models and clinically (Baguley and Andersson, 2007; Hammill and Campbell, 2018).

*In vivo* assessments of hearing functionality via auditory brainstem response (ABR) and postmortem cochlear histology are common in AG animal models, albeit with methodological inconsistency and variable reports of hearing dysfunction (Huth et al., 2011; Ogier et al., 2020). Longenecker et al. (2020) recently expanded on these traditional hearing assessments by showing that the acoustic startle reflex (ASR), a reflexed-based behavioral assay (Davis, 1984; Gómez-Nieto et al., 2020), can identify potential AG-induced changes to the central auditory system. Amikacin was also shown to induce behavioral evidence of hyperacusis, a sensitivity to loud sounds, and tinnitus, a ringing or buzzing in the ears (Longenecker et al., 2020), which has been observed in noise-induced (Auerbach et al., 2014; Shore and Wu, 2019) and salicylate-induced tinnitus animal models (Salvi et al., 2021). It is unknown if these auditory dysfunctions are specific to amikacin treatment or if they are a general side effect of AG antibiotics. Tinnitus is a prevalent side effect of many classes of drugs which include non-steroidal anti-inflammatory drugs, antibiotics, cancer drugs, diuretics, antimalarial drugs, and antidepressants. Indeed, a recent meta-analysis of clinical reports from the Cochrane library shows that tinnitus is one of the more common side effects for CF patients with recurrent AG treatments (Smith et al., 2018). Hyperacusis is under-reported clinically but is known to be debilitating and comorbid with tinnitus (Pienkowski et al., 2014), increasing with the number of years a patient has experienced tinnitus (Raj-Koziak et al., 2021; Refat et al., 2021). Thus, it is important for AG-induced auditory dysfunctions to be further investigated in animal models to develop potential therapeutics for these diseases of maladaptive plastic change.

Ebselen, a novel anti-inflammatory and anti-oxidant drug, has shown clinical efficacy for ameliorating hearing loss caused by acute noise exposure, and chronic Meniere’s disease, and is now investigating therapeutic interventions for tobramycin-induced ototoxicity (Kil et al., 2021). Less is known as to whether the anti-inflammatory properties of ebselen can prevent or treat diseases of the brain (Martini et al., 2019; Sharpley et al., 2020), but it is likely that inflammation plays an important role in AG-induced auditory dysfunctions (Jiang et al., 2017b; Wood and Zuo, 2017). Encouragingly, recent work in mice has demonstrated that ebselen can reduce suprathreshold ABR wave fluctuations and hyperacusis caused by amikacin treatment (Longenecker et al., 2020). Therefore, ebselen, with its variety of proposed anti-oxidant/anti-inflammatory mechanisms of action (Wang et al., 2020), is a good candidate to investigate as a potential therapy for both peripheral and central sensory diseases.

Here we show the effects of a 14-day course of tobramycin in CBA/Ca mice. Small magnitude temporary threshold shifts were observed in many mice. The prevalence of tobramycin-induced hyperacusis and tinnitus as assessed by ASR was highest 2 weeks after treatment and observed to decrease over time. This corresponded to an increase in brain activity as measured by ABR amplitudes. In an amikacin model, ebselen provided statistically significant protection against tobramycin-induced threshold shifts and demonstrated some efficacy for protecting against tobramycin-induced tinnitus (Longenecker et al., 2020). Finally, we suggest potential mechanisms that could explain the AG-induced auditory dysfunctions we have observed in tobramycin and amikacin treated mice.

## Materials and Methods

### Subjects

**FIGURE 1:**
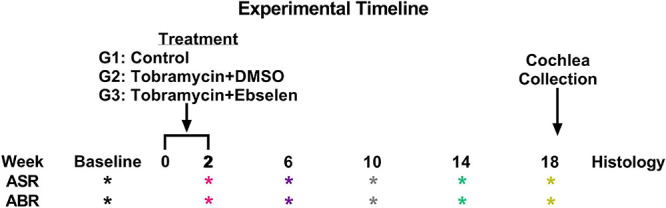
Experimental timeline. ASR and ABR tests (* symbol) are color coded to specific weeks the tests occurred after the start of treatment. This color code is maintained throughout the manuscript. Tobramycin treatment was carried out from week 0 to 2 for two separate dosing groups. Animals were sacrificed after week 18 tests for cochlear histology.

A total of 36 male and female CBA/Ca mice 3-6 months of age (at the start of experiments) were used in this study. Mice were born in house from parents obtained from Jackson Laboratories. Mice were housed 3 - 5 to a cage within a colony room with a 12-h light-dark cycle at 23°C. Hearing sensitivity and behavior was tracked longitudinally for each animal in a repeated measures design and animals were sacrificed for cochlear histology 18 weeks after the start of dosing following the final behavioral tests (see Figure 1).

### Drug Formulation, Dosing, and Schedule

Stock ebselen powder was dissolved in pure dimethylsulfoxide (DMSO) at 20 mg/ml and stock tobramycin was dissolved in distilled water at 50 mg/ml and both were stored at minus 20°C. Mice were divided into three groups: Group 1 (*n* = 8) served as a control and did not receive tobramycin or ebselen. Group 2 (*n* = 14) received the vehicle i.p. and tobramycin (200 mg/kg body weight diluted in 0.2 ml saline) s.c. thirty minutes later. Group 3 (*n* = 14) received ebselen i.p. (20 mg/ml in DMSO at 20 mg/kg body weight was diluted in fresh 0.5 ml sterile saline) and tobramycin at s.c. thirty minutes later. The daily dosing schedules for Groups 2 and 3 was identical and was continued for 14 days. During the dosing period, the health and condition of animals were monitored by body weight, which is known to decrease during AG treatments.

### Auditory Brainstem Response

Mice were anesthetized with isoflurane. Basal body temperature was maintained using a Gaymar T-pump warming pad set to 37°C and the animals’ health was monitored by observation of respiration and circulation. Each ear was otoscopically inspected prior to insertion of ear tips (Nicolet Biomedical, Inc.) for sound delivery. Monaural closed field ABRs (Intelligent Hearing Systems) were collected before (baseline), as well as at weeks 2, 6, 10, 14, and 18 from the start of AG treatment. Subdermal platinum needle electrodes (Grass Telefactor, Inc.) were placed with the active electrode at the vertex and the reference electrode to the test ear, and the ground to the contralateral ear. Each ear was tested independently. Stimuli consisted of pure tone pips (5 ms duration, rectangular envelope) at 4, 8, 16, and 20 kHz presented for 800 repetitions (19.3 r/s) at sound levels from 80 dB SPL to 0 dB SPL (initially 20 dB steps until near threshold, then 5 dB steps) calibrated with a 0.25-inch microphone (Brüel and Kjaer, 4939). Thresholds were measured in 5 dB increments and defined visually by the presence of the most robust peak (I or III) that was reliable within 0.1 ms. Thresholds were analyzed by a scientist blind to treatment and isolated from data collection.

### Behavioral Assessments of Hyperacusis, Tinnitus

#### Acoustic Startle Hardware/Software

Startle Reflex Hardware was purchased from Proxima Centauri Technologies (Julian, California). Each startle cabinet was lined with Sonex anechoic foam to minimize sound reflection and wave canceling sound echoes (Longenecker and Galazyuk, 2012). Sound levels from each cabinet’s speakers was calibrated with a 0.5-inch microphone (Brüel and Kjaer 4939). Startle Waveforms were recorded using load-cell platforms and calibrated with 100 g weights. Offline data processing with code written in visual basic was used to evaluate whether each trial was a startle or non-startle via template matching and startle magnitude data was converted from force to center of mass displacement (CMD) (Grimsley et al., 2015). Only legitimate startles were included and used in the final data analyses (Longenecker et al., 2018).

#### Input/Output Functions for Hyperacusis Assessments

Startle stimuli were pseudorandomly presented between 60- and 100-dB SPL in 5 dB steps. Intertrial intervals were randomized between 4 and 6 s. Each input/output (I/O) session lasted roughly 12 min and consisted of 135 total trials in which each startle intensity was presented 15 times. I/O assessments were collected before (baseline), as well as at weeks 2, 6, 10, 14, and 18 from the start of AG treatment. For each individual animal, hyperacusis was defined by a significant increase in startle magnitude (two-way ANOVA) between baseline and any time point after dosing.

#### GPIAS for Tinnitus Assessment

Gap prepulse inhibition of the acoustic startle reflex was used to assess behavioral evidence of tinnitus (Longenecker and Galazyuk, 2011; Longenecker and Galazyuk, 2016). The ability of mice to detect a gap of silence preceding a startle stimulus was determined by comparing the startle magnitude in response to a startle stimulus (white noise; 100 dB SPL) presented alone (SO) and a startle stimulus paired with a preceding (100 ms before) gap (20 ms long) of silence (GAP). Both trials were presented in a continuous narrowband noise carrier presented at 5 different frequencies (4, 8, 12.5, 16, 20 kHz) at a constant intensity of 65 dB SPL. Additionally, 15 startles presented in silence were used to monitor startle habituation. Intertrial intervals were randomized between 4 and 6 s. Eight mice were excluded from GPIAS analysis because their baseline gap detection ratios did not meet criterion.

A testing session was comprised of 15 blocks comprising 150 trials, lasting roughly 15 min. A block was defined by 10 trials containing 5 pseudorandom SO and GAP trials presented in a uniform carrier frequency. Throughout the session, each carrier frequency block was represented 3 times for a total of 45 trials. On each testing day, 3 GPIAS sessions were run on each mouse lasting roughly 45 min. The best performance ratio was used to determine an individual animal’s daily gap detection performance (Longenecker et al., 2018). GPIAS assessments were collected before (baseline), as well as at weeks 2, 6, 10, 14, and 18 from the start of AG treatment. For each individual animal, tinnitus was defined by a significant increase in the gap ratio (less inhibition) between baseline and any time point after dosing.

### Cochlear Histology

Following the final ABR and behavioral assessments, mice (∼6-12 months old) in three different groups (untreated control *n* = 8; tobramycin/DMSO *n* = 14; tobramycin/ebselen *n* = 14) were sacrificed with CO_2._ Cochlea were collected, fixed in 4% PFA overnight, then processed for whole mount immunostaining. After the bony wall was removed carefully, the intact membranous cochlea was isolated from the modiolus. After decalcification in 0.5 M EDTA for 1 h, the membranous cochlea was permeabilized and blocked in 0.2% Triton X-100, 1% BSA and 5% donkey serum in PBS. To assess damage to inner and outer hair cells, tissue of one cochlea from each group were incubated with primary antibody: Rabbit anti-Myosin VIIa (1:200 dilution) overnight at 4°C, rinsed in PBS, and incubated with secondary antibody: Alexa Fluor 594 Donkey anti-Rabbit IgG (1:500 dilution) for 2 h at room temperature. Then the tissue was stained with FITC-Phalloidin (1:1000 dilution) for 10 min. For ribbon synapse observation, tissue of the cochlea (untreated control n = 14; tobramycin/DMSO *n* = 25; tobramycin/ebselen n = 25) were incubated with two primary antibodies: Rabbit anti-GluR2 (1:500 dilution) and Mouse anti-CtBP2 (1:500 dilution) overnight at 4°C, rinsed in PBS, and incubated with two secondary antibodies: Alexa Fluor 594 Donkey anti-Rabbit IgG (1:500 dilution), Alexa Fluor 488 Donkey anti-mouse IgG (1:500 dilution) for 2 h at room temperature. After the immunostaining was finished, the membranous cochlea was cut at the apical turn and the basal turn, then further dissected, embedded in mounting media with DAPI. Samples were examined via an epi-fluorescent microscope (Nikon Eclipse Ti), images were captured via a charge-coupled device camera (Hamamatsu C11440). Hair cells were observed as row images of the cochlea and were captured under 20X plain lens, then single images were assembled to form the whole cochlea image by using Adobe Photoshop CC 2018. For ribbon synapse observation, raw images at 16 kHz region were captured under 60X oil optical lens with a Z-stack range at 6 – 10 μm (based on the distribution of ribbon synapse / inner hair cell), in 0.2 μm steps; raw images then deconvoluted and processed with maximum intensity project.

### Data Analysis

GraphPad Prism 9 was used for statistical analysis. One-way and two-way ANOVAs were used in data sets with normally distributed and equal sample sizes. Mixed models analyzed data that did not meet these assumptions. Sidak’s multiple comparison or Fisher’s LSD tests were used to discover individual differences at specific timepoints in the in vivo dosing study. Greyscale statistical symbols represent significance differences between dosing groups, while colored symbols represent specific significant differences between baseline and specific after dosing epochs. For each figure, the level of statistical significance is represented as: one symbol, p ≤ 0.05; two symbols, p ≤ 0.01; three symbols, p ≤ 0.001; four symbols, p ≤ 0.0001. Fisher’s exact test were used to analyze clinically relevant ABR threshold, hyperacusis, and tinnitus changes. To develop clinically relevant ABR threshold shift criterion, we modified ASHA guidelines for ototoxic change using pure tone audiometry (American Speech-Language-Hearing Association, 1994; Gu et al., 2020; Longenecker et al., 2020). Here, we identified ototoxic change using the following three criteria: (1) A ≥ 10 dB shift at three adjacent tested frequencies (4, 8, 16, 20 kHz). (2) A ≥ 15 dB shift at two adjacent tested frequencies. (3) A ≥ 20 dB shift at any one tested frequency. Each ear was tested and analyzed independently. The percentage of ears which met the threshold shift criteria was calculated for each time point (weeks 2, 6, 10, 14, and 18).

## Results

### Tobramycin Causes Temporary Threshold Shifts Without Permanent Cochlear Damage

**FIGURE 2:**
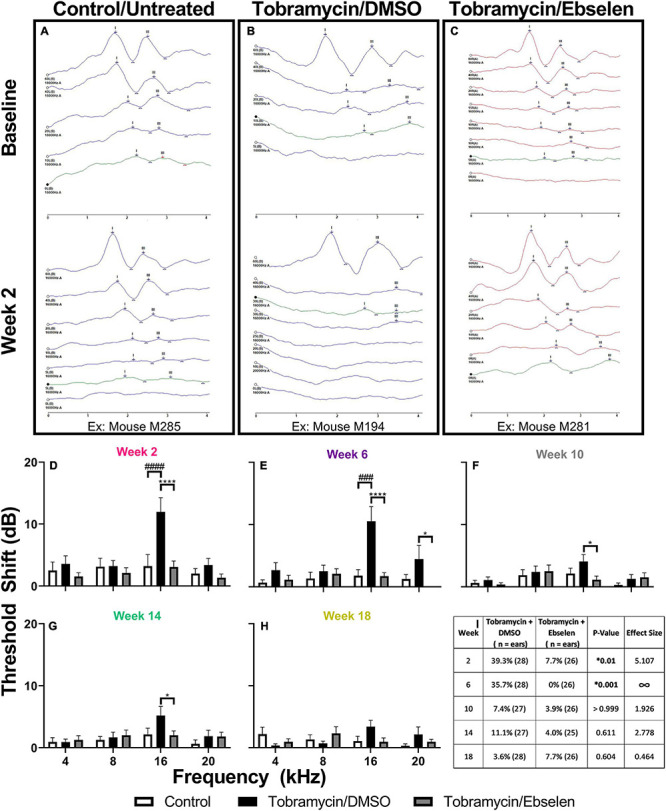
Evaluation of hearing loss caused by tobramycin. ABR traces from 16 kHz stimuli collected at baseline and 2 weeks after the start of treatment showing impact of tobramycin on hearing thresholds (**(A)**: untreated control, **(B)**: tobramycin/DMSO, **(C)**: tobramycin/ebselen). Blue and red traces show ABRs from left and right ears, respectively. Green trace in each panel represents threshold. Averaged tobramycin-induced ABR threshold shifts comparing testing groups at different epochs (**(D):** week 2, **(E):** week 6, **(F):** week 10, **(G):** week 14, **(H):** week 18) after the start of treatment. ABRs were collected for 4, 8, 16, and 20 kHz. Shifts represent the specific epoch minus the baseline ABR value for each group. Data is represented by threshold shift means and standard errors. Post hoc tests determined significant differences between testing groups, which are indicated as follows: # (black), between control and DMSO treated animals; # (gray), between control and ebselen treated animals; *, between DMSO and ebselen treated animals. **(I):** Clinically relevant change criteria table. (1) Criterion for CRC: A ≥ 20 dB shift at one frequency. (2) A ≥ 15 dB shift at two adjacent frequencies. (3) A ≥ 10 dB shift at three adjacent frequencies. Each ear was analyzed independently. The percentage of ears which met the ABR threshold shift criteria was calculated for each time point (weeks 2, 6, 10, 14, 18). Two-sided Fisher’s exact tests were used statistically evaluate differences between testing groups. The effect size represents relative risk (Koopman asymptotic score).

To examine the effects of tobramycin on hearing sensitivity, we documented ABR thresholds up to 18 weeks from the start of AG treatment. Following a standard 14-day tobramycin regimen, mean ABR thresholds were elevated 10-15 dB at 16 kHz from baseline levels (Figure 2). ABR traces from baseline and 2 weeks following the start of dosing are shown from an untreated control (Figure 2A), a tobramycin/DMSO treated (Figure 2B, and a tobramycin/ebselen treated mouse (Figure 2C). A two-way ANOVA found a significant effect of dosing between subjects at 2 weeks [F_(2, 265)_ = 9.724, *p* < 0.001] and 6 weeks [F_(2, 264)_ = 11.40, p < 0.001] after the start of dosing (Figures 2D,E). Significant effects for frequency were also found at 2 weeks [F_(3,265)_ = 5.363, *p* = 0.001], 6 weeks [F_(3,264)_ = 3.172, *p* = 0.025], 10 weeks [F_(3,262)_ = 3.499, *p* = 0.016] and 14 weeks [F_(3,257)_ = 2.820, *p* = 0.040] after dosing. Interactions between dosing and frequency were seen at 2 weeks [F_(__6, 265)_ = 3.118, *p* = 0.006] and 6 weeks [F_(6, 264)_ = 2.660, *p* = 0.016]. Šidák’s multiple comparison tests showed highly significant differences between control and tobramycin/DMSO groups at 16 kHz during the 2-week (Figure 2D, *p* = 0.001) and 6-week (Figure 2E, *p* = 0.001) epochs. Specific differences at 16 kHz were also seen between tobramycin/DMSO and tobramycin/ebselen groups at week 2 (*p* < 0.001), week 6 (*p* < 0.001), week 10 (*p* = 0.014), and week 14 (*p* = 0.021), as well as at 20 kHz at the 6-week epoch (*p* = 0.036) (Figures 2D-G). Tobramycin-induced threshold shifts were resolved by week 18 after the start of dosing (Figure 2H). To evaluate if these ABR deficits were significant on a per ear level, we applied the recently developed clinically relevant change criteria (Figure 2I; Gu et al., 2020; Longenecker et al., 2020). When evaluated with the Fisher’s exact test, these responder criteria revealed a significant difference between tobramycin/DMSO and tobramycin/ebselen treated ears at week 2 (39.3% vs 7.7%, *p* = 0.010) and week 6 (35.7% vs. 0%, *p* < 0.001), but not at later timepoints after dosing. These data suggest that although the mean threshold shifts were relatively mild after tobramycin treatment, a sizeable minority of mice experienced noticeable hearing impairment via a clinically relevant hearing assessment.

To investigate if the central auditory system became more reactive following tobramycin treatment, we assessed ABR wave III/wave I amplitude ratios (Supplementary Figure 1). We found that for most frequencies and amplitudes that there was a general increase in the wave III/wave I amplitude ratio (Supplementary Figures 1A-F) in tobramycin treated animals. While a significant effect of time was not found, a trend towards increased ratios over time was observed at some frequencies (reference to 100% dashed line). A between subjects mixed-effects analysis found an effect of dose for 16 kHz 40 dB ratios [F_(2, 69)_ = 3.295, *p* = 0.043], in which subjects of the tobramycin/DMSO group ratio was higher at most epochs than the subjects of the control or tobramycin/ebselen groups (Supplementary Figure 1F). Specific Fisher’s LSD post hoc significance values can be seen in Supplementary Table 1. The trends observed here closely resemble those seen in amikacin treated mice (Longenecker et al., 2020). Such changes in ratios could be explained by increases in wave III amplitudes or decreases in wave I amplitudes from baseline levels. We found that wave I amplitudes were mainly decreased, especially at higher frequencies (Supplementary Figures 2, 3). Additionally, some wave III amplitudes for 40 dB stimuli did increase (Supplementary Figure 2).

**FIGURE 3:**
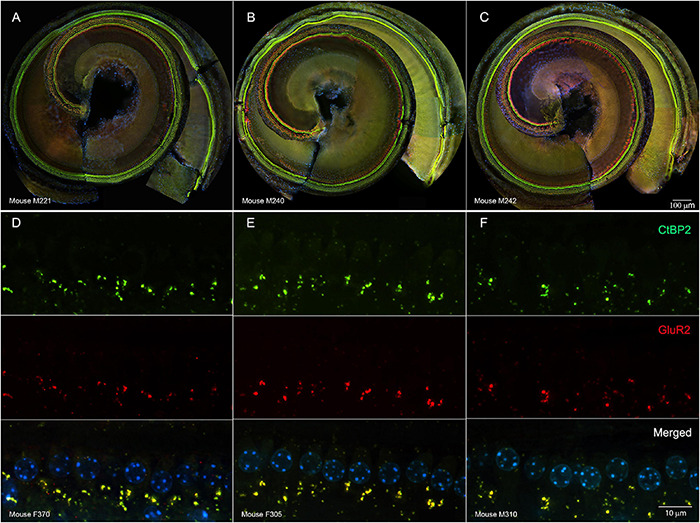
Representative whole mount cochlear images displaying cochlear hair cells for each treatment group (**(A):** untreated control, **(B):** tobramycin/DMSO, **(C):** tobramycin/ebselen [Scale bar = 100 μm]). No observable evidence of tobramycin-induced outer hair cell or inner hair cell loss. Outer hair cells were stained with anti-myosin VIIa antibody (red) and inner hair cells were stained with FITC phalloidin (green). Representative micrographs showing pre and post synaptic densities in the 16 kHz region for each treatment group (**(D):** untreated control, **(E):** tobramycin/DMSO, **(F):** tobramycin/ebselen [Scale bar = 10 μm]). When comparing treatment groups, no observable differences were apparent when examining the immunolabeled presynaptic marker CtBP2 (green), postsynaptic marker GluR2 (red), or the merged images (yellow), which include hair cell nucleus marker DAPI (blue).

Such changes electrophysiological changes were not readily explained by damage to cochlear sensory cells. In representative cochlear whole mounts, inner and outer hair cells remained intact from the base to the apex in untreated controls (Figure 3A), tobramycin/DMSO treated (Figure 3B), and tobramycin/ebselen treated animals (Figure 3C). At the region representative of 16 kHz hearing, no observable differences were seen in pre- (CtBP2 green) and post- (GluR2 red) synaptic densities between untreated controls (Figure 3D), tobramycin/DMSO treated (Figure 3E), and tobramycin/ebselen treated animals (Figure 3F). This was true throughout all the cochlea regions we analyzed which spanned all frequencies we examined in this study.

### Tobramycin Treatment Can Induce Hyperacusis and Tinnitus-Like Behavior

**FIGURE 4:**
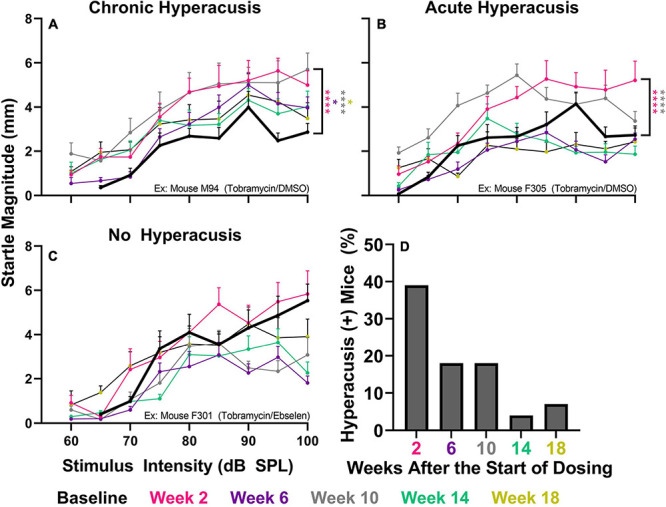
Evaluation of behavioral evidence of hyperacusis for individual animals. IO Startle stimulus/response functions recorded from 3 representative tobramycin-treated mice. **(A):** Example of a tobramycin-treated mouse with chronic hyperacusis, **(B):** Acute hyperacusis, and **(C):** no hyperacusis. Startle response magnitude CMD (mm) as a function of stimulus intensity (60 to 100 dB SPL) represent each specific epoch (color coded) compared to baseline (bolded black line). Data is represented by means and standard errors. *Post hoc* significant differences between specific epochs (color coded) and baseline are indicated as follows: *p ≤ 0.05; **p ≤ 0.01; ***p ≤ 0.001; ****p ≤ 0.0001. **(D):** Histogram representing the % of all tobramycin treated mice which showed statistically significant increases in startle IO functions at each epoch after dosing.

**FIGURE 5:**
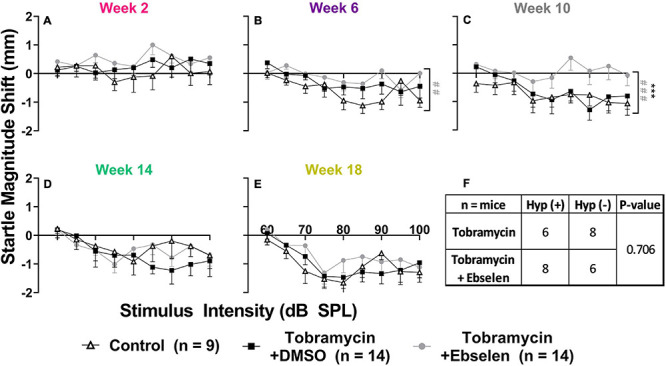
Evaluation of behavioral evidence of hyperacusis by dosing group. Averaged tobramycin-induced IO stimulus/response functions shifts comparing testing groups at different epochs (**(A):** week 2, **(B):** week 6, **(C):** week 10, **(D):** week 14, **(E):** week 18) after the start of treatment. Startle response magnitude CMD (mm) as a function of stimulus intensity (60 to 100 dB SPL) represent each specific epoch minus baseline. The value 0 on the y-axis represents no change from baseline startle reactivity. Data is represented by means and standard errors. Post hoc tests determined significant differences between testing groups, which are indicated as follows: # (black), between control and DMSO treated animals; # (gray), between control and ebselen treated animals; *, between DMSO and ebselen treated animals. **(F):** Contingency table for the development of hyperacusis which shows the number of mice which met or did not meet the criteria for hyperacusis (significant increase in startle IO function at any epoch when compared to baseline). Each animal was analyzed independently. Two-sided Fisher’s exact tests were used statistically evaluate differences between tobramycin dosed groups.

To investigate if tobramycin treatment resulted in amplified startle responses to loud acoustic stimuli as it did with amikacin treatment (Longenecker et al., 2020), we conducted ASR input/output assessments (Figures 4, 5). Following tobramycin treatment startle responses were greatly exaggerated in some mice, examples of which are seen in Figure 4. A two-way repeated measures ANOVA showed significance of time after treatment for mouse M94 [F_(3.715, 467.4)_ = 9.624, *p* < 0.001] and mouse F305 [F_(4.061, 441.0)_ = 34.84] (Figures 4A,B). For these mice, Dunnett’s multiple comparison tests revealed that startle responses were significantly elevated at week 2 (*p* < 0.001), week 6 (*p* = 0.037), week 10 (*p* < 0.001), and week 18 (*p* = 0.011) for mouse M94, as well as week 2 (*p* < 0.001) and week 10 (*p* < 0.001) for mouse F305. Such significant elevations in startle were not observed for example mouse F301 (Figure 4C). Time after dosing was also a significant factor for Mouse F301 [F_(4.526, 415.5)_ = 12.96, *p* < 0.001), but startle magnitudes significantly decreased over time, following the normal habituation to the startle stimulus. To tabulate the incidence of hyperacusis-like behavior following tobramycin treatment, the percentage of individual mice that demonstrated statistically significant increases in startle response for each epoch after dosing were evaluated with responder criteria (Figure 4D). At the week 2 epoch roughly 40% of mice experienced hyperacusis-like behavior, which fell to less than 10% by week 14. This responder analysis is important because it can elucidate that the symptoms of individual mice, which can be missed by group mean averages as seen in Figure 5. Between subject group analysis revealed very little hyperacusis-like behavior, as IO functions for each dosing group were below the baseline (below 0) startle magnitude at most epochs, representative of habituation (Figure 5). This was not surprising, because the majority of mice did not show hyperacusis-like behavior (Figure 4D). Two-way ANOVAs revealed that significant effect of treatment at week 2 [F_(2, 3__67__)_ = 3.139, *p* = 0.045], week 6 [F_(2,__311__)_ = 4.758, *p* = 0.009], and week 10 [F_(2,__304__)_ = 11.11, *p* < 0.001] (Figures 5A-C). Animals treated with tobramycin showed less habituation as a group, because a subset of these animals developed hyperacusis, while control animals did not (Figure 5). Šidák’s multiple comparison tests revealed that the tobramycin/ebselen treated group maintaining significantly higher levels of startle than the untreated control group at week 6 (*p* = 0.007) and the control group (*p* = 0.002) and DMSO group (*p* = 0.003) at week 10. By week 14 and 18 after the start of dosing, no significant differences were seen between groups with startle responses trending well below baseline startle reactivity (Figures 5D,E). To evaluate if increases in hyperacusis-like behavior were significantly different between treatment groups we conducted a Fisher’s exact test. Based on relatively similar mean startle response magnitude averages between groups (Figures 5A-E), it was unsurprising to see no significant difference between tobramycin/DMSO and tobramycin/ebselen groups (Figure 5F). Based on this dataset, ebselen did not play a major role in preventing the development of tobramycin-induced hyperacusis.

**FIGURE 6:**
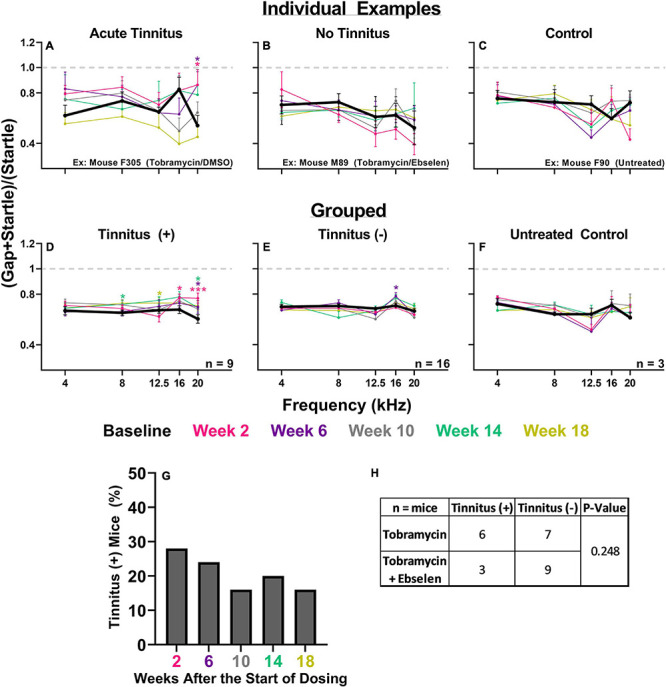
Evaluation of behavioral evidence of tinnitus for individual animals and dosing groups. **(A):** Example GPIAS functions for a tobramycin treated mouse with behavioral evidence of acute tinnitus, **(B):** a treated mouse with no tinnitus, and **(C):** an untreated control mouse. **(D):** GPIAS functions for the group mean averages of tinnitus mice, **(E):** tinnitus negative mice, and **(F):** untreated control mice. Tinnitus was defined as a significant GPIAS deficit at 1 or 2 adjacent frequencies at any epoch (color coded) compared to baseline (bolded black line). Data is represented by ratio means and standard errors. Post hoc significant differences between specific epochs (color coded) and baseline are indicated as follows: *p ≤ 0.05; **p ≤ 0.01; ***. **(G):** Histogram representing the % of all tobramycin treated mice which showed statistically significant gap detection deficits as described above at each epoch after dosing. **(H):** Contingency table for the development of tinnitus which shows the number of mice which met or did not meet the criteria for tinnitus. Each animal was analyzed independently. Two-sided Fisher’s exact tests were used statistically evaluate differences between tobramycin dosed groups.

To evaluate tobramycin-induced behavioral evidence of tinnitus, we used the GPIAS methodology which has been used to assess drug- and noise- induced tinnitus (Figure 6; Longenecker and Galazyuk, 2016; Longenecker et al., 2020). Individual examples of a mice that did and did not develop gap detection deficits following tobramycin treatment demonstrate the variability between mice (Figures 6A,B). A repeated measures mixed-effects model showed an effect of time after dosing on gap detection for mouse F305 [F_(3.749, 263.9)_ = 3.859, *p* = 0.006], but not mouse M089 [F_(4.553, 321.4)_ = 0.5342, *p* = 0.734]. Fisher’s LSD tests revealed frequency specific gap detection deficits (20 kHz) at week 2 (*p* = 0.018) and week 6 (*p* = 0.028) when compared to baseline gap detection for mouse F305 (Figure 6A). These sorts of deficits were not observed in example mouse M89 which was also treated with tobramycin (Figure 6B), nor mouse M90 which served as an untreated control (Figure 6C). To further investigate the overall effect of tobramycin on gap detection we examined the grand mean average of animals that developed tinnitus or those which did not (Figures 6D,F). In the tinnitus positive group, a two-way repeated measures ANOVA found an effect of time after tobramycin dosing (F_(__4.110, 164.4)_ = 2.943, *p* = 0.021), but not in the tinnitus negative group (F_(4.346, 325.9)_ = 1.199, *p* = 0.311), or the untreated controls (F_(2.799, 27.99)_ = 0.8964, *p* = 0.449). Fisher’s LSD tests revealed frequency specific deficits at 8 kHz (week 18: *p* = 0.038), 12.5 kHz (week 14: *p* = 0.041), 16 kHz (week 2: *p* = 0.026), and 20 kHz (week 2: *p* = 0.001, week 6: *p* = 0.014, week 14: *p* = 0.048) in the tinnitus positive group (Figure 6D). Interestingly, the tinnitus negative group also demonstrated one significant frequency at 16 kHz (week 6: *p* = 0.386). The incidence of tinnitus-like behavior (Figure 6G) demonstrated a similar pattern as hyperacusis-like behavior (Figure 4D), as it was highest at 2 weeks after tobramycin dosing and gradually decreased over time. When examining if ebselen had an effect on preventing tinnitus-like behavior (Figure 6H), a Fisher’s exact test did not find a significant effect (p = 0.248) but was more impactful than in hyperacusis-like behavior (Figure 5F).

## Discussion

In this study we demonstrated that a 14-day course of tobramycin can lead to hearing deficits, hyperacusis, and tinnitus in CBA/Ca mice, that may be clinically relevant. Ebselen was shown to be effective at preventing hearing loss and demonstrated non-significant evidence of preventing the development of tinnitus or hyperacusis in tobramycin treated mice. These deficits were observed in the absence of obvious cochlear hair cell loss or ribbon synapse degradation. These results are further discussed in context of previous preclinical and clinical studies investigating aminoglycoside-induced auditory dysfunction.

### Tobramycin-Induced Temporary Threshold Shifts Can Occur Without Permanent Cochlear Damage

Tobramycin has been known to cause variable magnitudes of temporary and permanent threshold shift following a single course in animal models (Gu et al., 2020; Ogier et al., 2020) and humans (Garinis et al., 2020; Harruff et al., 2020). Here we confirmed that a standard course of tobramycin treatment caused consistent, yet small significant mean threshold shifts of roughly 10-15 dB SPL at 16 kHz in a CBA/Ca animal model (Figure 2). After 18 weeks after dosing, these hearing deficits decreased and returned to near baseline levels (Figures 2D-H). The responder criteria for hearing loss demonstrated a similar pattern, in which the percentage of mice with a relevant shift decreased over time (Figure 2I). When comparing the effect size dosing factors, ebselen demonstrated protection against tobramycin-induced threshold shifts for the first 2 epochs after the start of dosing, but not at later time periods because threshold shifts were non-significant after this point (Figure 2). A similar finding was observed with the clinically modified or adopted responder criteria (American Speech-Language-Hearing Association, 1994). This finding was comparable to the effect of ebselen on amikacin treated mice (Longenecker et al., 2020), and provides evidence that ebselen or other otoprotective drugs might work at ameliorating AG-induced hearing loss in clinic (Avci et al., 2016; Fox et al., 2016; Dogan et al., 2017; El-Anwar et al., 2018; Apaydin et al., 2021). However, the threshold shifts seen here were drastically different than those found with a similar tobramycin dosing experiment in Swiss-Webster mice (Gu et al., 2020). While little is reported on the susceptibility of ototoxicity for most strains of mice, the CBA/Ca is well known for its relative resistance to noise- (Davis et al., 2001), and age- (Zheng et al., 1999; Peguero and Tempel, 2015; Liu et al., 2019), related hearing loss. When comparing these studies, it seems the CBA/Ca mouse has more resistant to AG-ototoxicity. However, the commercially available tobramycin used in each experiment could have differed by specific strain. This possibility was highlighted by an important recent finding which demonstrated that specific strains of gentamycin can have differential cochleotoxic effects (O’Sullivan et al., 2020). This could create a major confound in ototoxicity studies due to the variability of strain-induced damage and partially explains the variability of published results in the literature (Ogier et al., 2020). Nevertheless, a future study should investigate a tobramycin ototoxicity/dose response curve for CBA/Ca mice.

Just as we and others have discovered, aminoglycosides are not acutely ototoxic after one course of using non-lethal doses in most species and strains of laboratory animals (Longenecker et al., 2020; Ogier et al., 2020). In fact, cochlear hair cell loss was minimal or absent following one 14-day course of tobramycin (Figures 3A-C). It was also shown that spiral ganglion neurons and their synapses remain intact, at least after a recovery period of 16-18 weeks after tobramycin treatment (Figures 3D-F) or amikacin treatment (Longenecker et al., 2020). It is possible that immediate or early changes in synapse integrity following tobramycin treatment could explain the temporary auditory dysfunction documented in this study (Liu et al., 2015; Hong et al., 2018). Indeed, potentially relevant but largely non-significant increases in ABR wave III/wave I amplitude ratios (Supplementary Figure 1) suggest either central compensation from altered peripheral input (Gold and Bajo, 2014), or direct effects of AGs on the central nervous system (Segal et al., 1999; Abd-Elhakim et al., 2021).

### Hyperacusis and Tinnitus Appear After Tobramycin Treatment

**FIGURE 7:**
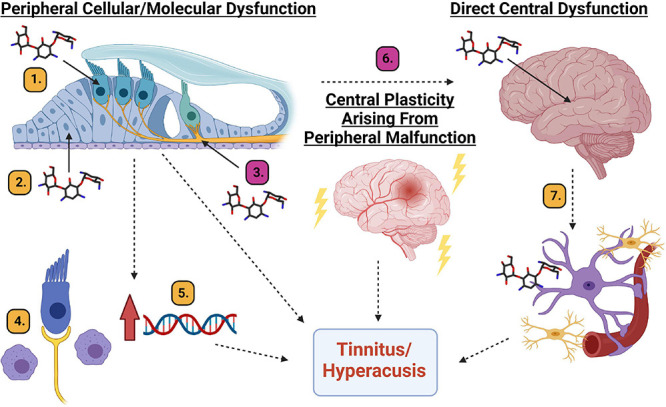
Diagram of potential mechanistic explanations for AG-induced tinnitus and hyperacusis. Peripheral cellular or molecular dysfunction: (1). Hair cell cellular dysfunction. (2). Supporting cell dysfunction. (3). Spiral ganglion neuron/synapse dysfunction. (4). Inflammation induced macrophage activation. (5). Upregulation of genes responsible for inflammation. Central plasticity arising from peripheral malfunction. (6). Changes in the neural code related to maladaptive plastic changes to the auditory system. Direct central dysfunction. (7). Aminoglycoside crossing the blood brain barrier leading to inflammation and cellular/molecular responses from microglia and astrocytes which can result in changes to neural firing patterns. Magenta # mechanisms are partially supported by data from this manuscript and Longenecker et al., 2020. Yellow # mechanisms require further scientific exploration. Created with BioRender.com.

The most important finding of this study is that tobramycin can cause behavioral signs of hyperacusis and tinnitus in mice (Figures 4, 5, 6). Additionally, these abnormal behaviors occur in the presence of only a mild loss of hearing sensitivity. These new findings are consistent with prior findings that amikacin can cause these behavioral symptoms following a mild threshold shift, although the mechanism will need to be elucidated (Longenecker et al., 2020; Figure 7). The largest difference between the two studies was that tobramycin treated mice demonstrated less startle magnitude increases after treatment than amikacin treated mice (Figure 5 vs. Figure 6 from Longenecker et al., 2020). This was especially true at the early epochs following treatment (Figures 5A-C). Tobramycin led to 50% and amikacin led to 82.3% of mice developing behavioral signs of hyperacusis. In both studies, hyperacusis-like symptoms were highest at epochs tested near the start of dosing and decreased thereafter (Figure 4D). The majority of mice treated with amikacin showed drastic increases in startle magnitude in contrast to a minority of mice treated with tobramycin. However, it is important to note that hyperacusis manifested differently between mice. Increased input/output startle functions such as the presented example of tobramycin treated mouse M94 (Figure 4A) was more commonly observed in amikacin treated mice (Longenecker et al., 2020). M94 demonstrated consistent hyperacusis throughout the testing period while F305 only experienced hyperacusis at weeks 2 and 10 after the start of dosing followed with clear habituation the rest of the epochs (Figures 4A,B). This temporal fluctuation disparity explains the dramatic difference in effect size between these mice which were both treated with tobramycin. Due to the lower incidence and startle magnitude shifts in tobramycin treated animals, it is not surprising that there was a non-significant effect of ebselen in preventing tobramycin-treated hyperacusis (Figure 5F). The effect sizes of dosing between epochs remained low throughout the study. The likely explanation of this phenomenon is that hyperacusis can be observed despite the natural response for mice to habituate to the startle reflex over time (Figures 4B,C, 5). Long-term habituation of the ASR is a universal phenomenon across multiple species and testing conditions (Davis, 1984) and has been specifically observed several strains of mice (Bullock et al., 1997; Pilz et al., 2014; Longenecker et al., 2018). This fact amplifies the importance of the odd behavioral shift towards dramatic increases in startle magnitude seen in some tobramycin (Figures 4A,B), and amikacin treated mice (Longenecker et al., 2020). The main reason hyperacusis was not observed in group averages of tobramycin treated mice (Figure 5) is because most mice did not develop hyperacusis and habituated to the startle (Figures 4D, 5F), which therefore decreased the average startle magnitude (Figures 5A-E). Amikacin treatment led to a higher incidence and magnitude of startle response shift, which explains the more elevated treatment group averages previously seen (Longenecker et al., 2020). Taken together, data from these studies suggest that aminoglycosides can dramatically increase startle responses in some animals despite habituation.

Behavioral evidence of tinnitus was also observed in tobramycin treated animals (Figure 6), although it is important to note the limitations of the GPIAS method in the context of AG-cochleotoxic experiments which were discussed in detail previously (Longenecker et al., 2020). We found that 9 out of 25 mice (36%) experienced gap detection deficits indicative of tinnitus at some point during the 18 weeks of testing after treatment, and similar to hyperacusis and hearing loss (Figures 2, 4D), the incidence decreased over time (Figure 6G). AG-induced tinnitus is thought to have an incidence rate between 12 and 37.5% in CF or tuberculosis patients (Daud et al., 2014; Harruff et al., 2020; Sabur et al., 2021). The other interesting point to note is that tobramycin treatment caused consistent gap detection deficits. When tinnitus-positive animals were grouped together, a significant broad-frequency deficit in gap detection was observed at multiple epochs (Figure 6D). The greatest deficits were seen at high frequencies (i.e. 20 kHz), which is higher than the frequency with the greatest threshold deficits (16 kHz), a common report following noise exposure in animal studies (Turner et al., 2012; Coomber et al., 2014; Longenecker and Galazyuk, 2016). Importantly, when averaged together, animals determined to be tinnitus negative had a gap detection deficit at 16 kHz at week 6 (Figure 6E). This deficit might be due to loss of hearing sensitivity at 16 kHz rather than a tinnitus percept at this frequency (Figure 2). Although not statistically significant, ebselen co-treatment trended towards the prevention of the tinnitus-like behaviors (Figure 6H).

As discussed above, hyperacusis was found to be more prevalent than tinnitus following tobramycin treatment which is consistent with what has been reported after amikacin treatment (Longenecker et al., 2020). Clinically, the incidence rates of these maladies are correlated, but not 1:1 (Baguley and Hoare, 2018; Cederroth et al., 2020). A major confound is that clinical studies are nearly entirely self-reported and based on patient reported outcomes over the last week or month. Large clinical studies have found a significant positive correlation between tinnitus and hyperacusis (Cederroth et al., 2020; Raj-Koziak et al., 2021). Another study found that hyperacusis percept shows a positive correlation for the persistence of tinnitus (Refat et al., 2021). It is likely that the neural code and anatomy for these maladies have unique but overlapping pathways, which could explain the results from these AG studies (Eggermont, 2021). A recent report demonstrated that hyperacusis was developed in rats to a non-damaging noise exposure of 70 dB SPL (Thomas et al., 2019). This study also showed that hearing sensitivity as assessed by ABR and light and fluorescence cochlear histology are grossly intact after AG treatment, suggesting that hyperacusis can be developed in the absence of significant cochlear cell loss (Thomas et al., 2019; Longenecker et al., 2020). A critical point to make when comparing amikacin and tobramycin studies is that there might be a link between behavior and electrophysiological measures of AG-induced auditory dysfunction. Amikacin treated mice demonstrated significant increases in ABR amplitude wave III/wave I ratios along with higher incidence and magnitude of hyperacusis-like behavior (Longenecker et al., 2020). Here we report lower incidence and magnitude rates of hyperacusis-like behavior which correlated to less significant increases in ABR ratios (Figure 4 and Supplementary Figure 1). Future studies should evaluate if this correlation between behavior and brain physiology reveals an underlying mechanism of tinnitus and/or hyperacusis (Figure 7). However, an important validation of this animal work was seen in CF-patients with lifetime histories of AG use (Westman et al., 2021). The Westman et al. (2021), study demonstrated that AG use was correlated with enhanced acoustic reflex growth functions compared to those with less or no AG exposure. The authors speculate that this finding more likely explains a central mechanism of auditory dysfunction, like hyperacusis or tinnitus, rather than cochlear related dysfunctions.

### Towards a Mechanistic Understanding of AG-Induced Tinnitus and Hyperacusis

In reviewing data from this tobramycin study, and the prior amikacin study (Longenecker et al., 2020), with the recent human study (Westman et al., 2021), it seems that hyperacusis and tinnitus might be common side-effects of AG treatment. The mechanism(s) to explain these AG-induced symptoms need to be investigated and could be explained in three unique, or more likely, overlapping pathways: peripheral dysfunction (cellular or molecular), central plasticity resulting from peripheral dysfunction, or direct central disfunction (Figure 7).

It is thought that all types of cells in the cochlea can be affected by aminoglycosides (Jiang et al., 2017a, b). Hair cells are known to be vulnerable to AGs and many other drugs (Figure 7(1), but at clinically relevant doses, it is unlikely that AGs lead to significant hair cell damage in animals or humans (Figure 3; Ogier et al., 2020). Even if hair cell death were observed, the resulting auditory dysfunction would not be fluctuating or diminishing over time (Figures 2, 4, 6; Longenecker et al., 2020), as hair cell loss is permanent. Alternatively, cochlear supporting cells, actively phagocytize dying hair cells, and expand into the deficit, and maintain epithelial/ionic integrity (Figure 7(2); Bird et al., 2010; Monzack et al., 2015) as well as release anti-inflammatory proteins (May et al., 2013). These cells need to be studied in more detail to ascertain if they are important in mediating or modulating AG-induced auditory dysfunction. Evidence supporting the hypothesis that SGNs and/or their synapses undergo temporary degradation or remodeling following AG exposure has been observed (Figure 7(3); Liu et al., 2015; Hong et al., 2018). This hypothesis is further supported by data showing decreases in ABR wave I amplitudes following AG treatment, especially at high frequencies (Supplementary Figures 2, 3). As mentioned above, when considering the wave III/wave I ratio, a temporary increase was observed (Supplementary Figure 1; Longenecker et al., 2020), because wave III amplitudes increased slightly while wave I decreased. Another likely mechanism for tinnitus and hyperacusis might increase inflammation in the cochlea (Jiang et al., 2017b). It has been shown that AGs cause inflammation in the cochlea and induce macrophage recruitment/activation which may modulate cochleotoxicity (Figure 7(4); Sato et al., 2010; Wood and Zuo, 2017; Hirose and Li, 2019). Lastly, it has been shown that AGs can trigger altered gene expression in several pathways including JNK, NF-κB, stress response, apoptosis, cell cycle control, and DNA repair pathways, or modulate ion channels responsible for signal transduction (Figure 7(5); Tao and Segil, 2015; Jiang et al., 2017b). Transient cochlear inflammation leading to macrophage recruitment and changes in gene expression changes may elicit the upstream dysfunctions marked by the behavioral symptoms of tinnitus and hyperacusis.

Central maladaptive plastic changes in response to peripheral insult has become central dogma in the field of tinnitus (Figure 7(6); Roberts et al., 2010; Langguth et al., 2013; Auerbach et al., 2014; Henton and Tzounopoulos, 2021). Data from animals and humans have found many unique but likely overlapping mechanisms to explain the neural plastic changes related to tinnitus which include: increases in spontaneous (non-sound evoked) firing rate neural activity (Longenecker and Galazyuk, 2011; Berger et al., 2014; Galazyuk et al., 2019); increased incidence of neurons burst firing (Ma et al., 2006; Bauer et al., 2008), increased or decreased neural synchrony (Eggermont and Tass, 2015; Marks et al., 2018), thalamocortical dysrhythmia (De Ridder et al., 2015), altered balance between excitatory and inhibitory neurotransmission (Middleton et al., 2011; Llano et al., 2012; Ma et al., 2020; Zhang et al., 2021), cortical reorganization (Engineer et al., 2011; Jeschke et al., 2021), and neural inflammation (Fuentes-Santamaría et al., 2017; Wang et al., 2019; Deng et al., 2020). The mechanisms of hyperacusis are much less studied but are thought to have similar etiologies as tinnitus (Auerbach et al., 2014). A recent study found that hyperacusis-like behavior correlated to enhanced sound-driven firing rates and reduced first spike-latencies of auditory brainstem neurons (Martel and Shore, 2020). These findings corroborate the mechanistic explanation of hyperacusis observed in models of tobramycin, amikacin, and salicylate, that neural gain increases in the auditory neuroaxis (Supplemental Figure 1; Longenecker et al., 2020; Salvi et al., 2021). Central plasticity following any changes to the cochlea are likely, and thus represent a candidate mechanism for explaining the behavioral evidence of tinnitus and hyperacusis seen here (Figures 4-6).

To what extent do AGs cross the blood brain barrier and cause direct neural dysfunction (Figure 7(7)? Some evidence suggests it is indeed occurring in animals and humans. Recent reviews have described that aminoglycosides such as tobramycin and amikacin are neurotoxic (Grill and Maganti, 2011; Rezaei et al., 2018). Some of the proposed mechanisms for this neurotoxicity include: excitotoxic activation of NMDA receptors within the cochlea, oxidative stress, calcium channels modulation, and localized inflammatory response. Auditory brainstem neurons in a chicken model were shown to decrease in size and then recover after 10 days of gentamicin treatment (Lippe, 1991). A similar set of studies in guinea pigs found that neurotropin-3 could protect spiral ganglion neurons from AG-induced degeneration (Ernfors et al., 1996; Duan et al., 2000). In mice, AG-induced ABR ratio amplitude increases were observed with tobramycin and amikacin (Figure 4, 5, 6; Longenecker et al., 2020) which suggests a clear trend towards increased neural activity in the auditory brainstem (Auerbach et al., 2014; Roberts and Salvi, 2019). Recent work has shown that brain inflammation can lead to tinnitus-like behavior in animals (Fuentes-Santamaría et al., 2017; Wang et al., 2019; Deng et al., 2020). If so, then ebselen would be a prime candidate for mitigating potential AG-induced inflammation (Kalinec et al., 2017; Jiang et al., 2017b; Wood and Zuo, 2017), as it has been implicated as a powerful anti-inflammatory and neuroprotective therapeutic (Wang et al., 2020). AG-penetration of the blood brain barrier in humans is understudied, however it has been suggested that AGs might be particularly neurotoxic in patients with compromised blood brain barriers (Segal et al., 1999), a condition which has been observed in CF patients (Vaughn et al., 1996).

### Future Studies to Evaluate AG-Cochleotoxicity and Otoprotective Strategies

Recent evidence indicates that a single course of IV tobramycin can lead to hearing loss in CF patients (Garinis et al., 2020; Harruff et al., 2020) and that this loss of hearing sensitivity results in poorer speech discrimination (Harruff et al., 2020). More clinical studies need to document and confirm these results. Physiological examinations such as the ABR or middle-ear muscle reflex should also investigate if ABR wave III/wave I amplitude ratios are altered in patients taking AGs as they have been in noise/age related hearing loss investigations (Bharadwaj et al., 2019; Guest et al., 2019; Refat et al., 2021). Preclinical work should investigate the neurotoxic effects of AGs using clinically translatable dosing schedules (Longenecker et al., 2020), to parallel what has been reported in AG treated patients (Segal et al., 1999; Daud et al., 2014; Harruff et al., 2020; Sabur et al., 2021). As discussed above, the CBA/Ca mouse would make a good model for researching this question because of its good hearing sensitivity throughout its lifespan. Furthermore, mice with blood brain barrier vulnerabilities should be tested against CBA/Ca mice to investigate if AGs can cause a higher incidence of hyperacusis and tinnitus. These sorts of studies would benefit from neurological recordings from auditory nuclei as well as brain histology to investigate the direct effects of AGs on neural anatomy and physiology. Additionally, AG strain differences should be examined to see if all strains of common AGs like tobramycin, amikacin, and gentamycin result in differential cochleotoxic effects (O’Sullivan et al., 2020). This could lead to safer AG treatments and prevent many of the permanent side-effects. Future studies should continue to evaluate therapeutic interventions for AG cochleotoxicity. To improve translatability, future experiments should test if ebselen or other anti-inflammatory drugs can provide additional relief from cochleotoxic side-effects with extended dosing beyond the 14 day-AG treatment (Hammill and Campbell, 2018). If so, such pharmaceuticals could prevent or even treat the chronic ototoxic side-effects of AG treatment, especially in CF.

## Data Availability Statement

The raw data supporting the conclusions of this article will be made available by the authors, without undue reservation.

## Ethics Statement

The animal study was reviewed and approved by Institutional Animal Care and Use Committee at Sound Pharmaceuticals, Inc.

## Author Contributions

RL, RG, and JK designed research. RL, RG, and JH performed research. RL and RG analyzed data. RL and JK wrote the manuscript. All authors contributed to the article and approved the submitted version.

## Conflict of Interest

The authors disclose that they are employed by Sound Pharmaceuticals and have stock ownership in the Company. The authors declare that this study received funding from Sound Pharmaceuticals, Inc. The funder had the following involvement with the study: provided full financial support for this study. The funder was not involved in the study design, data collection and analysis, or decision to publish. All authors declare no other competing interests.

## Publisher’s Note

All claims expressed in this article are solely those of the authors and do not necessarily represent those of their affiliated organizations, or those of the publisher, the editors and the reviewers. Any product that may be evaluated in this article, or claim that may be made by its manufacturer, is not guaranteed or endorsed by the publisher.

## Abbreviations

AG: aminoglycosides
CF: cystic fibrosis
SGN: spiral ganglion neurons
ABR: auditory brainstem response
ASR: acoustic startle reflex
kHz: kilohertz
DMSO: dimethyl sulfoxide
GPIAS: gap-induced prepulse inhibition of the acoustic startle reflex
GAP, trial with gap preceding startle SO: trial with startle only
ANOVA: analysis of variance
IO: input/output
CMD: center of mass displacement.
