# Supplementary material for: Development of Tinnitus and Hyperacusis in a Mouse Model of Tobramycin Cochleotoxicity
Source: Front Mol Neurosci. 2021 Sep 1;14:715952. doi: 10.3389/fnmol.2021.715952 (PMC8440845; doi:10.3389/fnmol.2021.715952)

Supplementary Material

Supplemental Figure/Table Legends:

Supplemental Figure 1. ABR wave III/I amplitude ratios show trend towards increasing after tobramycin treatment when dosing groups are compared. Averaged ABR Wave III/I ratios (%) from 60- (A, C, E, G) and 40-dB (B, D, F, H) SPL stimuli collected at 4, 8, 16, and 20 kHz, comparing groups at baseline and each epoch after AG treatment (weeks 2, 6, 10, 14, 18). Date is represented by ratio means and standard errors. Notice the values higher than the dashed grey line (reference for wave III/wave I 1:1 ratio). Post hoc significance values are posted in Supplemental Table 1 below.

Supplemental Table 1. Post hoc significance levels and p-values for Supplemental Figure 1. Significant differences between testing groups are indicated as follows: #, between control (C) and tobramycin/DMSO (D) treated animals; #, between control and tobramycin/ebselen (E) treated animals; *, between tobramycin/DMSO and tobramycin/ebselen treated animals.

Supplemental Figure 2. ABR wave I and wave III amplitudes (peak to peak) shifts for 40 dB stimuli. Averaged ABR wave I (A, C, E, G) and wave III (B, D, F, H) amplitude shifts from 40-dB SPL stimuli collected at 4, 8, 16, and 20 kHz. Shifts represent the specific epoch minus the baseline ABR value for each group (weeks 2, 6, 10, 14, 18). Data is represented by threshold shift means and standard errors.

Supplemental Figure 3. ABR wave I and wave III amplitudes (peak to peak) shifts for 60 dB stimuli. Averaged ABR wave I (A, C, E, G) and wave III (B, D, F, H) amplitude shifts from 40-dB SPL stimuli collected at 4, 8, 16, and 20 kHz. Shifts represent the specific epoch minus the baseline ABR value for each group (weeks 2, 6, 10, 14, 18). Data is represented by threshold shift means and standard errors.

Supplemental Figure 1:

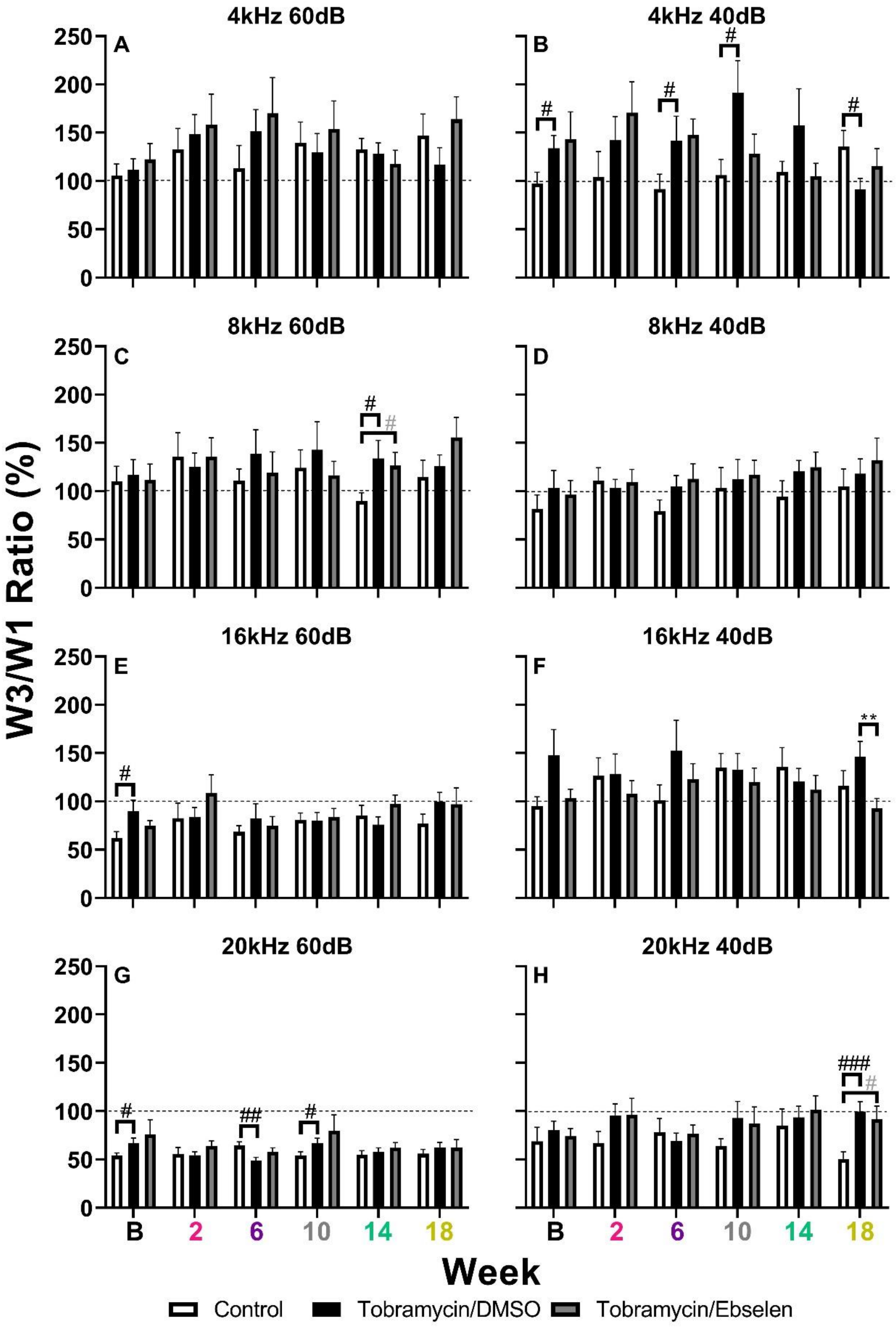

Supplemental Table 1:

Panel	Comparison	Epoch	Sig Level	P value
B	C vs D	B	#	0.0488
B	C vs D	6	#	0.0179
B	C vs D	10	#	0.0288
B	C vs D	18	#	0.0360
C	C vs D	14	#	0.0411
C	C vs E	14	#	0.0308
E	C vs D	B	#	0.0384
F	D vs E	18	**	0.0068
G	C vs D	B	#	0.0293
G	C vs D	6	##	0.0047
G	C vs D	10	#	0.0446
H	C vs D	18	###	0.0005
H	C vs E	18	#	0.0137

Supplemental Figure 2:

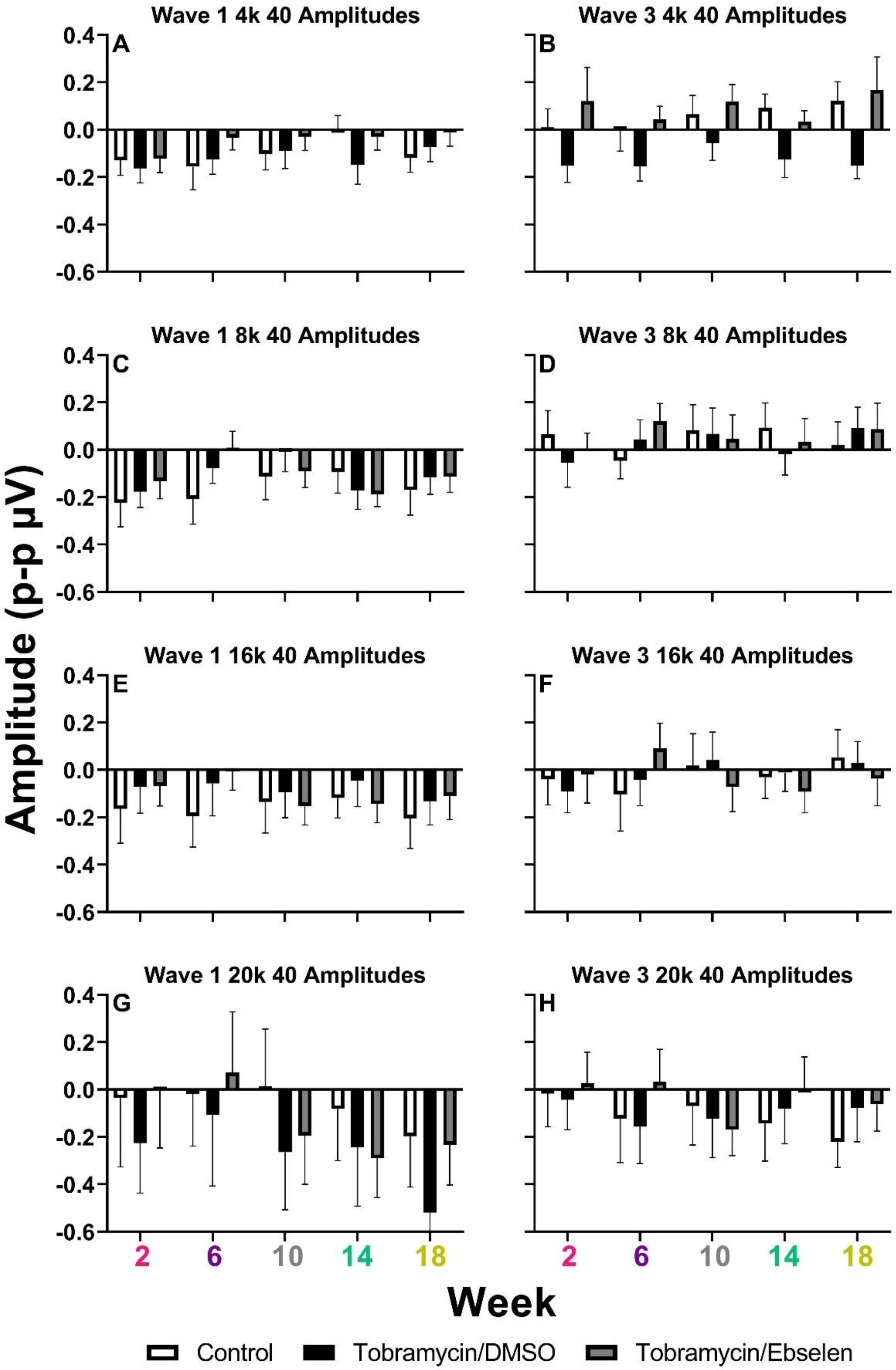

Supplemental Figure 3:

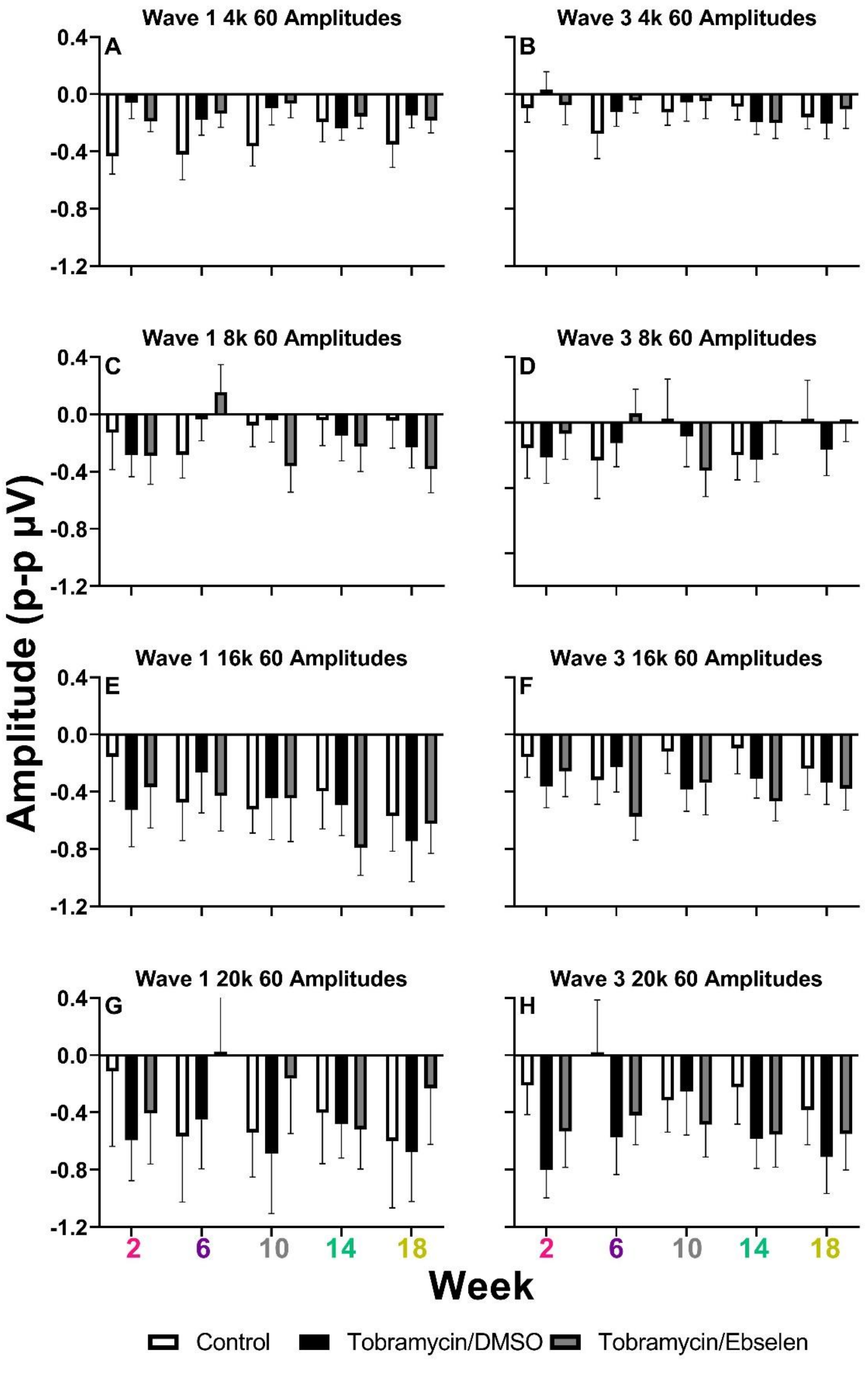

Supplement: Supplementary file 1 [file Data_Sheet_1.PDF]
